# miRNA484 and miRNA335-5p as Potential Diagnostic Biomarkers for CA19-9-Negative Pancreatic Ductal Adenocarcinoma

**DOI:** 10.3390/biom16081160

**Published:** 2026-08-10

**Authors:** Nayra Oliveira Prado, Anelis Maria Marin, Carolina Zem, Micheli de Marchi, Diogo Dias Araújo, Maria José Ferreira Alves, Miyuki Uno, Roger Chammas, Dalila Lucíola Zanette, Mateus Nóbrega Aoki

**Affiliations:** 1 Laboratory of Molecular Oncology, Carlos Chagas Institute, Oswaldo Cruz Foundation (Fiocruz), Curitiba 81310-020, Brazil; nay_antares@hotmail.com (N.O.P.); czem@aluno.fiocruz.br (C.Z.); michelidemarchi27@gmail.com (M.d.M.);; 2 Center for Translational Research in Oncology (LIM24), Instituto do Cancer do Estado de São Paulo (ICESP), Hospital das Clínicas da Faculdade de Medicina da Universidade de São Paulo (HCFMUSP), São Paulo 01246-000, Brazilmaria.jfalves@hc.fm.usp.br (M.J.F.A.); miyuki.uno@hc.fm.usp.br (M.U.);; 3 Comprehensive Center for Precision Oncology (C2PO), Universidade de São Paulo, São Paulo 01246-000, Brazil

**Keywords:** pancreatic ductal adenocarcinoma, circulating miRNAs, early diagnosis, CA19-9, plasma biomarkers, small RNA sequencing, miRNA484, miRNA335-5p, liquid biopsy

## Abstract

Pancreatic ductal adenocarcinoma (PDAC) is one of the most lethal cancers worldwide, largely because it is diagnosed late. Diagnosis typically relies on imaging and biopsy, but only after tumors have advanced, which reduces treatment effectiveness. Current biochemical biomarkers are limited to CA19-9, which is more useful for prognosis and monitoring than for diagnosis. Circulating miRNAs may offer minimally invasive complementary biomarkers for PDAC diagnosis, potentially accounting for ethnic and treatment-related variables. We performed small RNA sequencing on plasma from five non-metastatic-stage and five metastatic-stage PDAC patients and 10 healthy controls to generate an exploratory miRNA profile. Candidate miRNAs were then validated by RT-qPCR in an independent cohort of 70 PDAC patients and 106 healthy controls, and nine miRNAs showed promising differential plasma expression. Notably, miRNA484 and miRNA335-5p were associated with PDAC in CA19-9-negative patients, with ROC AUCs of 0.984 and 0.88, respectively. Overall, these results indicate that miRNA484 and miRNA335-5p may have clinical value as complementary biomarkers for PDAC patients who are CA19-9-negative.

## 1. Introduction

Pancreatic cancer is among the most aggressive and lethal malignancies worldwide, with nearly 510,000 new cases and 467,409 deaths in 2022, and a higher incidence in developed regions [1]. Approximately 90% of pancreatic cancers are pancreatic ductal adenocarcinomas (PDACs), which arise from ductal cells and often remain asymptomatic until they obstruct the pancreatic duct, producing nonspecific symptoms such as jaundice, nausea, and weight loss [2]. Despite advances, the 5-year survival for PDAC remains very low –13.3% in the USA for 2015–2021—and is lower in many other regions [3,4]. Diagnosis by imaging is challenging because of the pancreas’ location and the typically small tumor size at early stages, which leads to a high proportion of patients being diagnosed with advanced disease. Minimally invasive biomarkers are limited; the glycoprotein CA19-9 is the most widely used marker but has restricted diagnostic utility and is more useful for prognosis and disease monitoring [5,6]. However, the diagnostic performance of CA19-9 remains suboptimal, as a considerable number of patients with PDAC have negative CA19-9 levels regardless of disease stage, including both metastatic and non-metastatic disease [7,8]. Additionally, CA19-9 will be falsely negative in Lewis’s antigen-negative individuals [7,8]. Consequently, the discovery of complementary biomarkers is crucial to address this unmet clinical need.

MicroRNAs (miRNAs) are small noncoding RNAs of ~20–25 nucleotides in mature form that regulate mRNA translation and influence many tumor-related processes. Aberrant miRNA expression is often associated with a complex set of alterations in cellular regulation. In PDAC, miRNAs actively contribute to disease progression by regulating gene expression involved in cell proliferation, apoptosis, invasion, and metastasis. Dysregulation of miRNAs directly influences multiple oncogenic pathways, including KRAS-related signaling [9,10]. Circulating miRNAs are detectable in diverse body fluids (plasma, urine, saliva, cerebrospinal fluid, and breast milk) and may be up- or downregulated in cancer, providing information on disease presence and progression. Furthermore, miRNAs are highly stable in biological fluids, as they are protected from RNase degradation when associated with protein complexes or encapsulated in extracellular vesicles. This stability reinforces their availability and supports their utility as minimally invasive biomarkers [11]. Despite their potential, the clinical application of circulating miRNAs remains challenging due to variability in sample processing, differences in detection platforms, and the lack of standardized normalization strategies, which contribute to inconsistency across studies, compromising reproducibility and clinical application [12].

In this study, we aimed to analyze plasma miRNAs from PDAC patients and healthy controls to identify differentially expressed miRNAs with potential diagnostic value. We performed small RNA plasma sequencing in non-metastatic stage (early-stage) and metastatic stage (late-stage) PDAC patients and matched healthy controls, followed by RT-qPCR validation. Of note, the non-metastatic group consisted of T2–T3 tumors and therefore does not correspond to truly early-stage disease (T1). Our results support the potential of circulating miRNAs as complementary biomarkers for PDAC diagnosis in Brazilian patients, especially for negative CA19-9 PDAC. This finding is particularly relevant from a clinical perspective, as the limited sensitivity of CA19-9 remains a major challenge in the diagnosis of pancreatic cancer. Therefore, the identification of alternative and complementary biomarkers is essential to improve PDAC diagnosis.

## 2. Materials and Methods

### 2.1. Study Design and Participants

This prospective case–control study was conducted between July 2022 and December 2024. Blood samples from pancreatic adenocarcinoma patients were collected at the São Paulo Cancer Institute (ICESP), São Paulo, Brazil, following the ICESP Biobank protocol, and healthy control samples were collected at Hospital do Trabalhador, Curitiba, Brazil. Both sites were approved by local ethics committees, as described in the Institutional Review Board Statement. All the participants provided written informed consent. Inclusion criteria for PDAC patients were age ≥ 18 years, histologically confirmed pancreatic adenocarcinoma (CID25), and clinical fitness for blood collection as determined by the treating team. For the discovery phase all the patients were treatment naïve at the time of sampling. For the validation phase, no restrictions were applied for tumor stage. Clinical data was collected upon availability. Health controls had no personal history or current suspicion of cancer. Age and sex distributions were matched between groups.

### 2.2. Discovery RNA Sequencing

For the discovery phase, 20 plasma samples were analyzed (10 PDAC and 10 healthy controls). The PDAC samples included 5 non-metastatic stage and 5 metastatic cases (classified by presence of metastasis at diagnosis and sample collection). Total RNA, including small RNAs, was extracted from plasma using the miRNeasy kit (QIAGEN; Hilden, Germany). Libraries were prepared using Illumina TruSeq Small RNA sample preparation protocols. Library quality and quantification were assessed with an Agilent 2100 Bioanalyzer High Sensitivity DNA chip (Agilent; Santa Clara, CA, USA). Single-end 50 bp sequencing was performed on an Illumina HiSeq 2500 (Illumina, San Diego, CA, USA) according to the manufacturer’s instructions.

### 2.3. RNA Seq Data Analysis

Raw reads were processed with the in-house ACGT101 miR pipeline (LC Sciences; Houston, TX, USA) to remove adapter dimers, low complexity reads, common RNA families (rRNA, tRNA, snRNA, and snoRNA) and repetitive sequences. Unique reads of 18–26 nt were mapped to precursor sequences in miRBase v22.0 by BLAST version 2.17.0 to identify known miRNAs and candidate novel 3p/5p derived miRNAs. Alignments allowed length variation at both 3′ and 5′ ends and one internal mismatch. Sequences mapping to annotated mature miRNAs were classified as known miRNAs. Sequences mapping to the opposite arm of known precursors were considered candidate novel 5p/3p miRNAs. Remaining mapped pre-miRNAs were BLASTed against reference genomes to determine their genomic locations. Unmapped sequences were BLASTed to genomes and potential hairpin structures were predicted from 80 nt flanking sequences using RNAfold [8]. Secondary structure criteria were: bulge nucleotides in stem ≤ 12; stem base pairs ≥ 16; folding free energy ≤ −15 kcal/mol; hairpin length (stems + loop) ≥ 50 nt; loop length ≤ 20 nt; bulge nucleotides in mature region ≤ 8; biased errors in a single bulge ≤ 4; biased bulges in mature region ≤ 2; errors in mature region ≤ 7; base pairs in mature region ≥ 12; and ≥80% of mature sequence located in the stem.

### 2.4. Differential Expression Analysis

Differential expression of miRNAs was performed by the DESeq2 v1.48.2 software (Bioconductor, R Foundation for Statistical Computing, Vienna, Austria) using normalized deep sequencing counts. Statistical tests (Fisher’s exact test, chi-square 2 × 2 or *n* × *n* tests, Student’s *t*-test, or ANOVA) were applied according to experimental design. False discovery rate (FDR) was applied and miRNAs with *p* below 0.05 and absolute fold change ≥ 2 were considered differentially expressed. Differentially expressed miRNAs were then subjected to enrichment analysis of GO (Gene Ontology) functions and KEGG pathways.

### 2.5. Plasma RNA Extraction for Validation Phase

Before miRNA extraction, 1 µL of synthetic cel-miRNA39-3p (1 nM) was added as an exogenous spike into each 100 µL of plasma. Total RNA was then isolated using the MagMAX™ mirVana™ Total RNA Isolation Kit (Thermo Fisher Scientific; Waltham, MA, USA) following the manufacturer’s protocol for serum/plasma.

### 2.6. cDNA Synthesis and RT qPCR

Reverse transcription was performed with the TaqMan Advanced miRNA cDNA Synthesis Kit (Thermo Fisher Scientific; Waltham, MA, USA) according to the manufacturer’s instructions. Quantification was performed using TaqMan miRNA assays and TaqMan™ Fast Advanced Master Mix (Thermo Fisher Scientific; Waltham, MA, USA) on a QuantStudio 5 real-time PCR system (Thermo Fisher Scientific; Waltham, MA, USA). The exogenous cel-miRNA39-3p (Thermo Fisher Scientific; Waltham, MA, USA) spike-in served as a normalizer to correct for miRNA extraction due to biochemical differences between the plasma samples. All qPCRs were run in duplicate with non-template controls.

### 2.7. Statistical Analysis

Relative expression was calculated using the 2^−ΔΔCT^ method [13]. qPCR data were normalized to the median Ct of the cel-miRNA39-3p spike in a single healthy control sample that was used as the calibrator. Statistical analyses and graphical representations were performed with GraphPad Prism 7.

## 3. Results

### 3.1. Discovery Phase

#### 3.1.1. Cohort Description

Ten treatment-naïve PDAC patients were selected for RNA sequencing and divided into two groups of five according to the absence (early PDAC) or presence (late PDAC) of metastasis at diagnosis (demographic and clinical data are shown in Table 1). Patient ages ranged from 35 to 84 years; the cohort was evenly sex balanced (50% male, 50% female). Blood samples were collected 16–86 days after diagnosis. Tumor location distribution was head 69.8%, body 22.6%, and tail 7.6%. The healthy control group consisted of 10 non-pancreatic cancer individuals, also with no evidence of any type of cancer, balanced in terms of age and gender, with no significant difference from the PDAC cohort.

#### 3.1.2. RNA Sequencing

**Figure 1:**
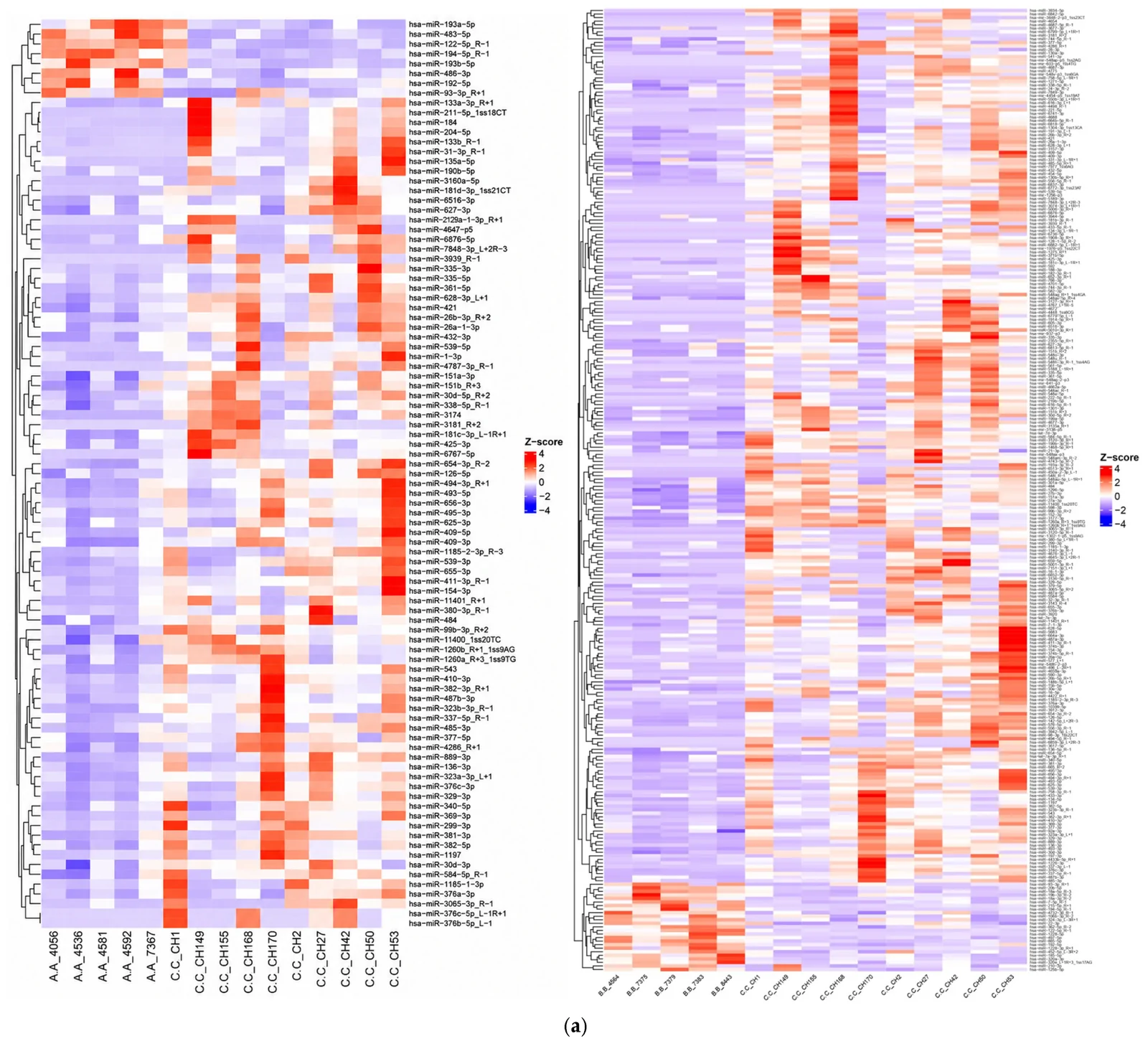

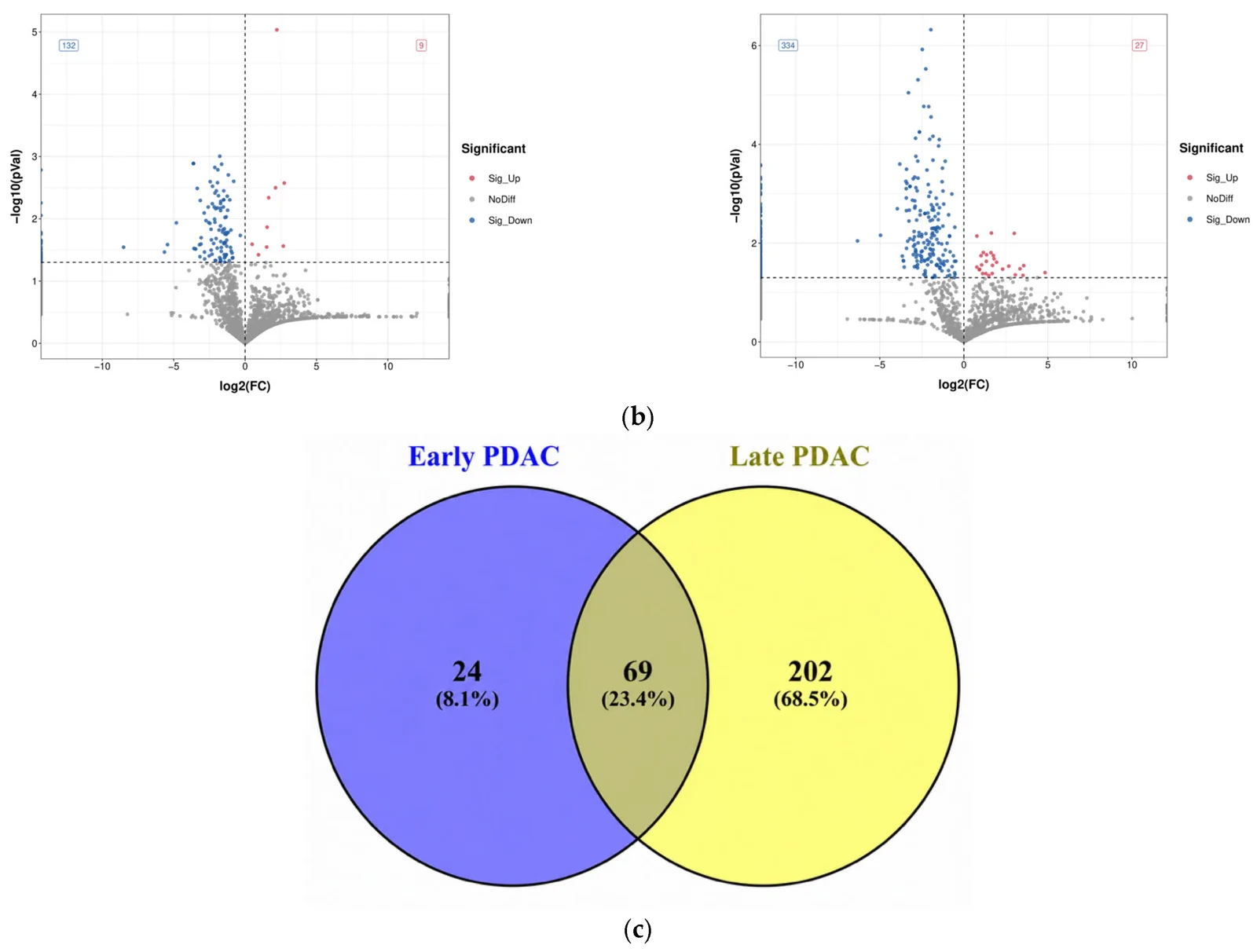
(**a**) Heatmap for miRNAs differentially expressed in non-metastatic PDAC vs. healthy controls (**left**) and metastatic PDAC vs. healthy controls (**right**), considering *p* < 0.05. The AA, BB and CC in x-axis represent non-metastatic PDAC, metastatic PDAC and healthy controls, respectively, and the following number represent the sample ID; (**b**) volcano plot for miRNAs differentially expressed in non-metastatic PDAC vs. healthy controls (**left**) and metastatic PDAC vs. healthy controls (**right**), considering *p* < 0.05, where blue dots represents miRNAs downexpressed in PDAC, and red dots miRNAs upexpressed in PDAC; and (**c**) Venn diagram for miRNAs with statistically significant different expression between PDAC and controls, considering *p* < 0.05.

miRNA sequencing was analyzed comparing non-metastatic PDAC versus healthy controls and metastatic PDAC versus healthy controls. RNA Seq quality metrics are provided in the Supplementary Figure S1 and Supplementary Table S1. Using a significance threshold of *p* < 0.05, and after removing sequences with non-human alignments (e.g., *Rattus norvegicus*, *Mus musculus*, *Cricetulus griseus*, *Callithrix jacchus*), 93 miRNAs were differentially expressed in non-metastatic PDAC versus healthy controls (8 upregulated and 85 downregulated), and 271 miRNAs were differentially expressed in metastatic PDAC versus healthy controls (25 upregulated and 246 downregulated). Of these, 24 miRNAs were altered only in non-metastatic PDAC, 202 were altered only in metastatic PDAC, and 69 were altered in both non-metastatic and metastatic PDAC. Figure 1a shows the heatmap for non-metastatic PDAC versus healthy controls on the left side and metastatic PDAC versus healthy controls on the right side. Figure 1b shows the volcano plot for miRNAs differentially expressed in non-metastatic PDAC vs. healthy controls on the left side and metastatic PDAC vs. healthy controls on the right side. Figure 1c presents a Venn diagram and stage-associated expression patterns. Differentially expressed miRNAs are listed in Supplementary Tables S2 and S3.

### 3.2. RT-qPCR Validation

**Figure 2:**
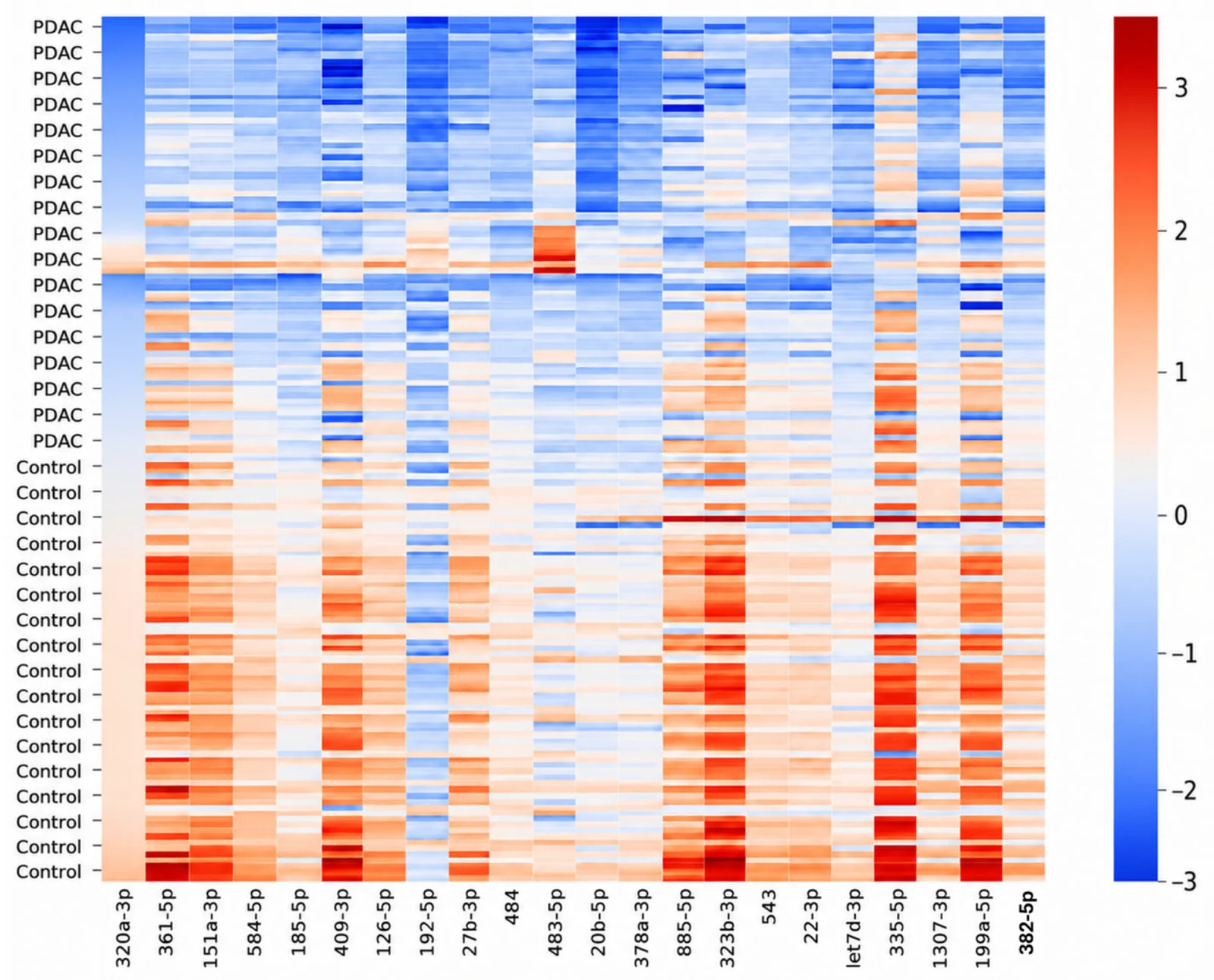
Heatmap for 22 miRNAs (represented on the x-axis) selected for validation with RT-qPCR in 70 PDAC patients and 106 healthy controls (represented on the y-axis).

**Figure 3:**
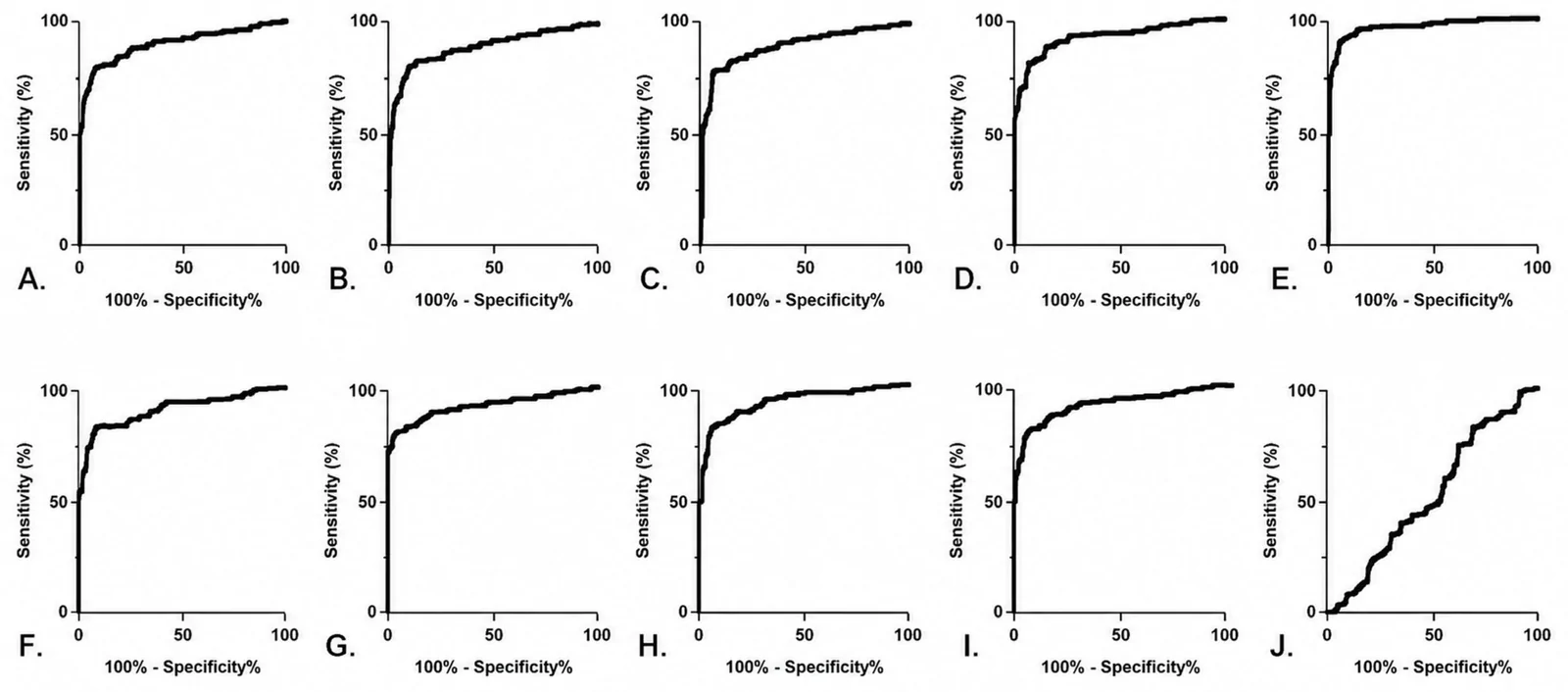
ROC curve for differentiation between PDAC patients and healthy controls for 9 selected miRNAs from (**A**–**I**), miRNA361-5p, miRNA409-3p, miRNA126-5p, miRNA27b-3p, miRNA484, miRNA323b-3p, miRNA543, let7d-3p and miRNA335-5p, respectively. Figure (**J**) corresponds to miRNA885-5p with an ROC curve of 0.533.

Candidate miRNAs for a primary independent validation cohort were selected when they showed high or medium expression levels in RNA sequencing to avoid technical limitations, showed a *p*-value < 0.05 for both the non-metastatic and metastatic PDAC samples in comparison with the healthy controls, showed the same trend in expression in both PDAC cohorts, and showed an increasing fold-change in metastatic PDAC in comparison to non-metastatic PDAC. Twenty-two miRNAs were selected for validation in 70 PDAC patients and 106 healthy controls using RT-qPCR. Table 2 summarizes the validation cohort characteristics and shows no significant differences between PDAC and healthy control groups for age, sex, or ethnicity. The healthy control cohort had a significantly higher body mass index, whereas a larger proportion of PDAC patients had diabetes. For those 22 selected miRNAs, 21 miRNAs were differentially expressed between PDAC and healthy controls in this primary independent validation cohort (Figure 2). With this finding, nine promising miRNAs that showed ROC curve AUC > 0.90 were selected as diagnostic candidates (Table 3, Figure 3): miRNA361-5p, miRNA409-3p, miRNA126-5p, miRNA27b-3p, miRNA484, miRNA323b-3p, miRNA543, let7d-3p, and miRNA335-5p. Despite statistically significant differences in expression between groups, other miRNAs showed ROC AUC lower than 0.90.

After that, the PDAC samples of the validation phase were separated by CA19-9 status as positive (>37 U/mL) and negative (<37 U/mL) when available, resulting in 37 and 15 positive and negative CA19-9 PDAC, respectively, and compared to 106 healthy controls. It was observed that four of the nine miRNAs showed different expression in both CA19-9 groups, as demonstrated in the table above (Table 4), while the remaining were differentially expressed just in the CA19-9-positive group, indicating that these four miRNAs may be a useful and accessory biomarker for PDAC with a CA19-9-negative result. The following table demonstrates the fold change for the expression of these four miRNAs in the PDAC CA19-9-positive and -negative samples when compared to a healthy control sample as a reference. The ROC AUC indicates the ability to differentiate the PDAC cohort and the healthy controls according to miRNA differential expression, where it can be observed that miRNA484 and 335-5p showed high ROC AUC in both the PDAC CA19-9-positive and -negative cohorts.

## 4. Discussion

PDAC is a complex disease, and its development is often clinically silent or presents with a wide range of nonspecific symptoms, which delay diagnosis and allow tumors to progress to advanced stages, complicating patient management and treatment [14]. Although obstructive jaundice due to common bile duct compression is a recognized symptom, it occurs in only about one-third of patients. Many diagnoses arise incidentally during routine examinations or follow-up of unrelated conditions, or after investigation of worsening diabetes; diagnostic workup typically includes CT and/or MRI and, subsequently, biopsy for staging [15].

In the present study, after RNA-seq analysis in the treatment-naïve PDAC patients in early and late stages and healthy controls, we identified nine circulating miRNAs with potential as diagnostic biomarkers for PDAC, among which miRNA484 and miRNA335-5p emerged as the most clinically relevant candidates due to their consistent differential expression in the CA19-9-positive and CA19-9-negative patients. These findings are particularly important considering the persistent diagnostic gap associated with CA19-9-negative PDAC cases.

A major challenge in PDAC is the lack of reliable biomarkers, and although CA19-9 remains the most widely used biomarker in clinical practice, its diagnostic performance is suboptimal, as 5–10% of the population lacks the Lewis antigen and is therefore unable to synthesize CA19-9, while a substantial proportion of confirmed PDAC patients present normal CA19-9 levels. Consequently, the false-negative results remain a significant clinical challenge, particularly in early-stage tumors. The identification of biomarkers capable of detecting these patients represents an unmet need in pancreatic cancer diagnostics [7,8,16]. MiRNAs can associate with proteins and multivesicular bodies and be released in exosomes, which are detectable in peripheral blood [7], and the literature reports numerous miRNAs and miRNA families associated with tumor initiation, progression, and prognosis. Dysregulation of these miRNAs contributes to carcinogenesis by repressing tumor suppressors [17,18] or activating oncogenic pathways [19,20,21,22]. However, only a subset of the described miRNAs has well-characterized molecular functions and pathways [23,24,25]. Several studies have proposed circulating miRNA signatures for PDAC detection, including EV-associated and plasma-derived biomarkers, frequently achieving sensitivities and specificities superior to conventional serum markers [23]. Collectively, these studies support the growing evidence that circulating miRNAs represent a robust and biologically relevant source of diagnostic information in PDAC.

Growing evidence indicates that miRNAs are active regulators of PDAC biology, as dysregulated miRNAs are involved in pathways associated with epithelial–mesenchymal transition, angiogenesis, proliferation, apoptosis, and chemoresistance. Since a single miRNA can simultaneously regulate multiple target genes, alterations in miRNA expression may exert broad effects on tumor progression, which directly contributes to the molecular heterogeneity observed in PDAC [12,26]. For example, miRNA21 is a widely reported oncogenic miRNA in cancer, such as pancreatic cancers, acting by silencing tumor suppressors and affecting proliferation, apoptosis, invasion, and migration [19,20,21,22], and miRNA155 is linked to chemoresistance [27]. Conversely, several miRNAs act as tumor suppressors, with let-7 family negatively regulating RAS and inhibiting proliferation; reduced let-7 expression is associated with worse survival [28,29], and miRNA34 family regulates p53 and MYC and is linked to apoptosis and reduced invasion.

Some PDAC studies report heterogeneous miRNA signatures, arising from methodological and biological differences. Variations in sample type (serum, plasma, tissue, or extracellular vesicles), in methods used for RNA isolation, sequencing platforms, normalization strategies, and patient selection criteria may considerably influence miRNA profiles. Moreover, differences in disease stage, treatment and tumor cellular composition may also contribute to inconsistencies between cohorts [17,27]. Concerning this, one conflicting and non-consensus issue is correlated to miRNA normalization for plasma quantification. While intracellular miRNA relative quantification by RT-qPCR is commonly normalized to internal housekeeping genes such as U6, snord RNAs, or specific miRNAs, plasma miRNA quantification is usually normalized to external spike-in targets. This external miRNA relies on a *C. elegans* sequence that is not present in humans and is used to assess plasma miRNA extraction across different plasma contents, normalize extraction efficiency, and serve as a data analysis normalizer.

Owing to the heterogeneity, recent efforts have focused on validating miRNA panels with high diagnostic accuracy using robust methods and well-defined cohorts. Makler & Asghar (2023) described a plasma exosomal panel (miRNA93-5p, miRNA339-3p, miRNA425-5p, miRNA425-3p) achieving 80% sensitivity and 94.7% specificity, and miRNA425-5p alone detected 100% of early-stage cases in that study [30,31]. Pu et al. (2024) used machine learning on EVs miRNA data and identified miRNA664a-3p as a primary marker; combined with CA19-9, it reached an AUC of 0.94 [30,32]. Potential applications include early diagnosis, prognosis prediction, therapeutic monitoring, and recurrence detection. It is important to note that miRNA-based biomarkers may be particularly useful in patients with negative (undetectable) or low CA19-9 levels, groups in which conventional biomarkers have limited diagnostic utility [17,26], as demonstrated in this manuscript.

Prospective studies, such as using the EPIC cohort collected years before diagnosis, show that the predictive accuracy of plasma miRNAs increases as diagnosis approaches [33]. In samples ≤2 years before diagnosis, miRNA10b, miRNA21-5p, miRNA30c, and miRNA106 b were higher in cases than controls; in the medium term (≤5 years), miRNA21-5p and miRNA30c were most strongly associated with risk, and miRNA10a, miRNA10b and miRNA30c remained significantly different even up to 12 years, suggesting early detectable alterations in tumorigenesis or predisposition [33,34].

Although the primary objective of this study was not to investigate molecular mechanisms, the biological functions previously attributed to miRNA484 and miRNA335-5p reinforce their biomarker potential. Both miRNAs are associated with pathways involved in proliferation, stemness, and tumor progression, suggesting that their altered circulating levels may reflect biologically meaningful events occurring during PDAC development. miRNA484 has been consistently described as a tumor suppressor in PDAC, with studies demonstrating that its downregulation is associated with activation of oncogenic pathways, including YAP/TEAD and Wnt/MAPK, which are involved in proliferation, survival, and tumor progression. Therefore, the reduced plasma levels observed in our cohort may reflect molecular alterations associated with pancreatic tumorigenesis [35,36]. Moreover, miRNA335-5p has also been reported as a tumor suppressor in PDAC, where experimental studies have shown that restoration of its expression reduces clonogenicity, invasiveness, and metastatic potential. Mechanistically, these effects have been associated with the regulation of stemness-related pathways, including OCT4 signaling. Interestingly, miRNA335 has previously been included in circulating miRNA signatures proposed for PDAC diagnosis, further supporting its relevance as a biomarker candidate [37,38].

Considering the findings, miRNAs miRNA484 and miRNA335-5p highlighted candidates, with significant differential expression between groups and consistent data in the PDAC patients with positive and negative CA19-9 levels, suggesting that these markers may be used as accessory diagnostic markers for CA19-9-negative PDAC patients. This accessory marker combination represents an effective approach to reduce false negatives, as demonstrated by miRNA panels combined with CA19-9 having better diagnostic performance compared to CA19-9 alone [33]. Our findings suggest that miRNA484 and miRNA335-5p may complement CA19-9 testing and contribute to reducing false-negative diagnoses in PDAC.

As a limitation, this study includes patients and healthy controls recruited from different institutions, which may have introduced bias in sample collection and processing. To minimize this effect, collection and processing protocols were standardized across all the participating centers. The blood samples were collected in EDTA tubes and processed under similar controlled conditions. Additionally, this study includes a limited cohort of PDAC patients in the discovery phase, largely due to the challenges associated with recruiting early-stage and treatment-naïve PDAC patients and correlated with financial limitations. This reduced discovery cohort may induce some bias for miRNA screening; however, this was mitigated with a larger and independent validation cohort. Also, as a primary independent cohort, more validation cohorts must be performed, especially considering the Brazilian ethnicity difference, where a miRNA-combined diagnostic model could be generated, associated with PDAC and with CA19-9 status. Unfortunately, stage distribution data were not available for all the patients in this validation cohort, as this information was missing from the medical records. However, we are currently expanding the cohort with new patients and actively collecting detailed staging data to further validate the identified miRNAs in future analyses. On this topic, the CA19-9-negative PDAC patients cohort serves as a rare cohort and is demonstrated here with a small sample size, with limited and careful findings and conclusions, requiring a more independent validation cohort. The absence of samples from patients with chronic pancreatitis, pancreatic cystic lesions, biliary obstruction, or other relevant diseases is an important limitation. Besides that, the plasma miRNAs here detected are not necessarily directly corresponding to PDAC tumoral tissue but may also be associated with the tumor environment and inflammatory cells; however, this does not compromise its role as a plasma biomarker. As well, biochemical plasma characterization will be improved in the following validation cohort, with quantification of molecules that may interfere with and bias miRNA expression such as glucose, lipid profile, hemoglobin, total bilirubin and total protein. In studies with small cohorts, ROC-derived metrics may be optimistic due to sampling variability and cohort-specific characteristics. This is a well-known limitation in biomarker discovery studies, where promising diagnostic performance in initial cohorts is frequently attenuated during external validation.

Regarding future perspectives, we understand that the combination of circulating miRNAs with other molecular biomarkers—such as circulating tumor DNA, proteomic profiles, and extracellular vesicle analysis—may help refine diagnostic accuracy and provide a broader understanding of PDAC biology. To gain a better understanding, in vitro functional assays may be conducted to elucidate as-yet-undescribed molecular mechanisms linking these miRNAs to PDAC tumorigenesis and progression processes. Another interesting approach relies on plasma miRNAs’ absolute quantification by dPCR, a technique that is not associated with a normalization process, such as *C. elegans* miRNA39-3p spike-in or housekeeping (U6, Snords, etc). Finally, advances in multi-omics approaches and machine learning may also favor the development of predictive models applicable in clinical routine, aimed at early detection and personalized treatment of pancreatic cancer.

## 5. Conclusions

In our study, comprehensive miRNA profiling by RNA sequencing provided targets for validation in a well-characterized cohort. We identified nine plasma miRNAs with diagnostic potential for PDAC in Brazilian patients and found four miRNAs that were significantly altered in the CA19-9-negative patients, highlighting miRNA484 and miRNA335-5p, indicating their promise as complementary screening markers. A key limitation is the relatively small PDAC sample in the validation cohort, reflecting difficulties in recruiting early-stage, treatment-naïve patients; independent and larger validations are therefore required. Larger, multi-center, prospective and mechanistic studies with stringent multiple-testing control and standardized pre-analytical procedures are needed to confirm clinical utility.

## Figures and Tables

**Table 1:** Demographic and clinical characteristics of patients.

Diagnostic Age	Diagnosis to Sample Collection (Days)	Sample Collection to Treatment (Days)	Gender	Self-Reported Ethnicity	Weight (kg)	Height (m)	BMI ^1^	Tumor Location	T	M	Neoadjuvant Therapy
58	16	NA	F	White	54	1.56	22.19	Head	2	0	No
67	41	5	F	White	60	1.53	25.63	Head	3	0	FOLFIRINOX
84	51	NA	M	Brown	42.3	1.38	22.21	Head	2	0	No
65	52	9	M	White	57	1.61	21.99	Head	3	0	FOLFIRINOX
54	69	14	F	White	77.5	1.88	21.93	Head	3	0	FOLFIRINOX
48	28	NA	M	Brown	68	1.69	23.81	Head	4	1	No
74	22	70	F	White	52	1.66	18.87	Head	4	1	Gencitabin
47	86	7	M	Brown	92	1.82	27.77	Body	4	1	FOLFIRINOX
35	40	123	F	White	52	1.57	21.10	Head	4	1	PanFLOX
59	70	2	M	Brown	60	1.78	19.00	Body	4	1	FOLFIRINOX

^1^ BMI: body mass index.

**Table 2:** Summary of full cohort characteristics and comparison between PDAC and control groups showing no significant differences in age, sex, or ethnicity.

		Gender (%)		Ethnicity (%)				
	Age	Male	Female	White	Black	Brown	BMI ^1^	Diabetes
PDAC	61.13 ± 1.32	47.55	52.45	54.91	13.72	31.37	22.16 ± 0.470	36.58%
Control	58.01 ± 0.96	42.03	57.97	62.26	14.16	23.58	26.78 ± 0.422 *	12.26% **

^1^ BMI: Body mass index; * *p* < 0.0001; ** *p* < 0.05.

**Table 3:** Nine miRNAs expression quantified by RT-qPCR in plasma of PDAC and healthy controls. The values for PDAC and healthy controls represent the mean fold-change compared to a reference healthy control sample, the standard deviation, the number of samples analyzed, and the ROC AUC. ROC: Receiver Operating Characteristic curve. AUC: area under the curve.

	361-5p	409-3p	126-5p	27b-3p	484	323b-3p	543	let7d-3p	335-5p
PDAC	0.93 ± 0.26 *n* = 70	0.46 ± 0.12 *n* = 60	0.67 ± 0.12 *n* = 70	0.49 ± 0.08 *n* = 70	0.32 ± 0.14 *n* = 70	0.65 ± 0.22 *n* = 58	0.85 ± 0.17 *n* = 63	0.37 ± 0.10 *n* = 68	0.58 ± 0.29 *n* = 61
Control	135.90 ± 31.14 *n* = 106	126.10 ± 32.42 *n* = 106	22.07 ± 4.15 *n* = 106	26.60 ± 5.39 *n* = 106	4.88 ± 0.68 *n* = 106	105.30 ± 31.82 *n* = 102	317.10 ± 74.22 *n* = 100	14.61 ± 3.26 *n* = 101	4.78 ± 0.73 *n* = 100
*p*-value	0.0004	0.0041	<0.0001	0.0001	<0.0001	0.0143	0.0009	0.0005	<0.0001
ROC AUC	0.9086 ± 0.0230	0.9190 ± 0.0211	0.9113 ± 0.0242	0.9346 ± 0.0204	0.9538 ± 0.02121	0.9131 ± 0.02239	0.9208 ± 0.0218	0.9212 ± 0.0236	0.9272 ± 0.02271

**Table 4:** Differential expression of four miRNAs in PDAC samples stratified by CA19-9-positive (>37 U/mL) and CA19-9-negative compared to healthy controls. Fold change (FC) is calculated by 2^−ΔΔCT^ using one healthy control sample as reference, as described in *Statistical analysis.* Sample size (N) represents the total number of samples in that cohort and target. AUC represents the Area Under the Curve for the ROC Curve when comparing the respective PDAC cohort (CA19-9-positive or CA19-9-negative) with healthy controls, while miRNA20b-5p showed a lower AUC and miRNA483-5p a weak AUC value.

	miRNA484			miRNA483-5p			miRNA20b-5p			miRNA335-5p		
	FC ^1^ (±Standard Error)	Samples	AUC ^2^ (Standard Error; 95% CI ^3^)	FC ^1^ (±Standard Error)	Samples	AUC ^2^ (Standard Error; 95% CI ^3^)	FC ^1^ (±Standard Error)	Samples	AUC ^2^ (Standard Error; 95% CI ^3^)	FC ^1^ (±Standard Error)	Samples	AUC ^2^ (Standard Error; 95% CI ^3^)
PDAC CA19-9-positive	0.39 ± 0.25 *	37	0.948 (0.025; 0.899 to 0.997)	61.93 ± 26.1 *	37	0.507 (0.068; 0.372 to 0.642)	0.45 ± 0.13 *	35 ***	0.849 (0.0395; 0.771 to 0.926)	0.21 ± 0.05 *	31 ***	0.948 (0.018; 0.911 to 0.984)
PDAC CA19-9-negative	0.11 ± 0.02 *	15	0.984 (0.009; 0.965 to 1.000)	58.77 ± 19.56 *	15	0.672 (0.110; 0.431 to 0.864)	0.49 ± 0.16 **	15	0.823 (0.055; 0.715 to 0.931)	0.66 ± 0.37 **	13 ***	0.88 (0.015; 0.839 to 0.949)
Healthy controls	4.88 ± 0.69	106		3.67 ± 0.69	106		3.06 ± 0.39	102 ***		4.78 ± 0.73	100 ***	

* *p* < 0.001; ** *p* < 0.05; *** cohorts with reduced number due to no target amplification. ^1^ FC: fold change compared to control; ^2^ AUC: area under the curve of the ROC curve; ^3^ CI: confidence interval.

## Data Availability

Dataset available upon request from the authors.

## Supplementary Materials

The following supporting information can be downloaded at https://www.mdpi.com/article/10.3390/biom16081160/s1: Figure S1: Pearson correlation for samples RNA-Seq replicate; Figure S2: Reads length size distribution, in base pair; Table S1: reads parameters for 10 RNA-Seq samples; Table S2: Different expressed microRNAs in early PDAC vs. healthy controls; Table S3: Different expressed microRNAs in late PDAC vs. healthy controls.
