# Supplementary material for: miRNA484 and miRNA335-5p as Potential Diagnostic Biomarkers for CA19-9-Negative Pancreatic Ductal Adenocarcinoma
Source: Biomolecules. 2026 Aug 10;16(8):1160. doi: 10.3390/biom16081160 (PMC13511162; doi:10.3390/biom16081160)
Supplement: Supplementary file 1 [file biomolecules-16-01160-s001.zip › biomolecules-4370771-supplementary.pdf]

Supplementary Table S1: reads parameters for 10 RNA-Seq samples

#Sample_ID	Total_Reads	Total_Bases	A%	T%	C%	G%	N%	Q20%	Q30%	GC%
A_4536	12481121	636537171	23,82	22,03	24,72	29,42	0,00	98,79	96,08	54,14
A_4581	10658816	543599616	23,74	24,12	22,72	29,42	0,00	98,99	96,58	52,14
A_4592	9221303	470286453	23,91	22,29	24,23	29,57	0,00	98,40	95,41	53,80
A_7367	10426159	531734109	23,77	23,89	22,85	29,48	0,00	98,70	95,78	52,33
A_4056	9321297	475386147	24,15	22,97	22,38	30,50	0,00	98,71	95,86	52,88
B_8443	7545213	384805863	23,66	22,20	22,21	31,93	0,00	98,52	95,28	54,13
B_7375	9818164	500726364	23,46	23,78	21,14	31,61	0,00	98,53	95,31	52,75
B_7379	10432859	532075809	22,80	23,66	22,99	30,54	0,00	98,66	95,54	53,53
B_7383	8407294	428771994	23,16	22,02	23,64	31,18	0,00	98,58	95,69	54,82
B_4564	8039564	410017764	23,63	22,07	24,44	29,86	0,00	98,51	95,49	54,30
C_CH1	10292180	524901180	23,38	24,56	22,08	29,98	0,00	98,96	96,43	52,06
C_CH2	11367243	579729393	23,70	23,81	23,09	29,40	0,00	98,87	96,35	52,48
C_CH168	8601986	438701286	24,01	23,02	23,73	29,23	0,00	98,56	95,24	52,97
C_CH170	10344943	527592093	23,15	22,85	24,15	29,84	0,00	98,74	96,03	54,00
C_CH149	9672950	493320450	23,44	23,22	24,12	29,22	0,00	98,66	95,74	53,34
C_CH155	15585544	794862744	22,64	24,34	22,49	30,53	0,00	98,79	96,24	53,02
C_CH42	9616964	490465164	23,68	23,17	23,92	29,24	0,00	98,79	95,94	53,15
C_CH50	9632437	491254287	23,95	23,49	23,86	28,70	0,00	98,64	95,71	52,56
C_CH27	14201666	724284966	23,39	23,66	23,44	29,51	0,00	98,84	96,09	52,95
C_CH53	17920422	913941522	24,05	24,13	23,33	28,49	0,00	98,81	96,28	51,81

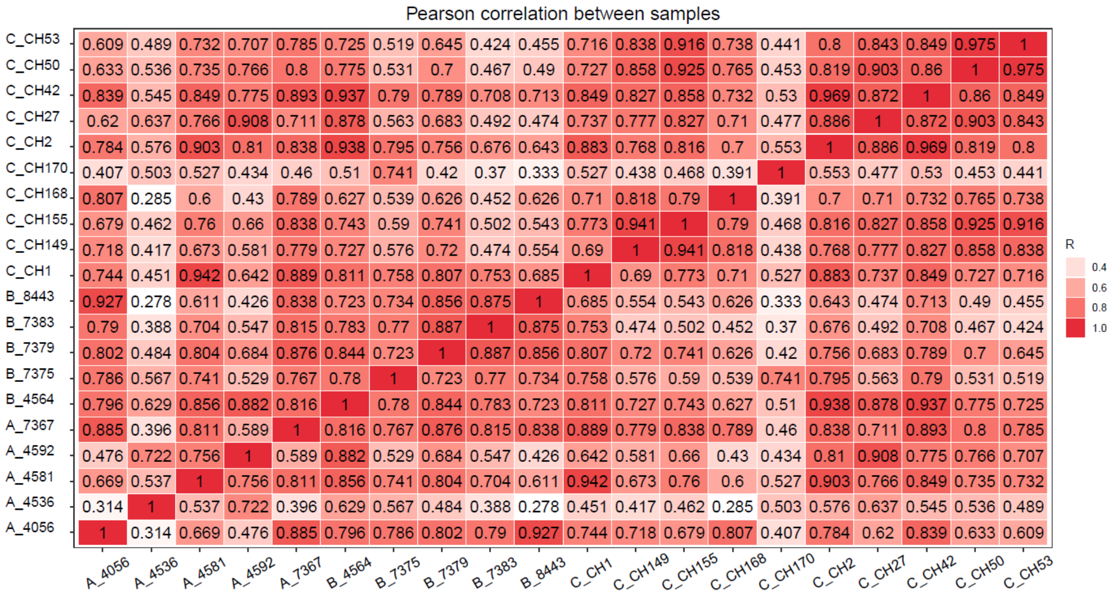

Supplementary Figure S1: Pearson correlation for samples RNA-Seq replicate

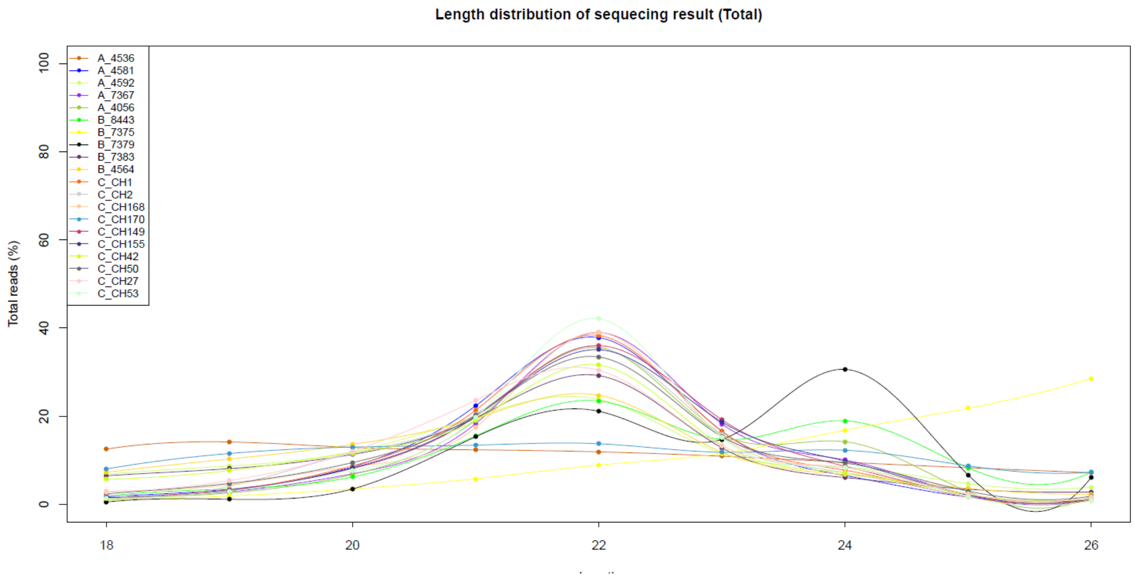

Supplementary Figure S2: Reads length size distribution, in base pair

Table S2: Different expressed microRNAs in early PDAC vs. healthy controls

ID	microRNA	Sequence	Expression	p value	Level
1	hsa-miR-122-5p_R-1	TGGAGTGTGACAATGGTGTTT	up	9,21E-06	high
2	hsa-miR-1260b_R+1_1ss9AG	ATCCCACCGCTGCCACCATT	down	9,89E-04	middle
3	hsa-miR-543	AAACATTCGCGGTGCACTTCTT	down	1,50E-03	middle
4	hsa-miR-628-3p_L+1	TTCTAGTAAGAGTGGCAGTCGA	down	1,63E-03	middle
5	hsa-miR-3174	TAGTGAGTTAGAGATGCAGAGCC	down	1,64E-03	middle
6	hsa-miR-421	ATCAACAGACATTAATTGGGCGC	down	1,98E-03	middle
7	hsa-miR-30d-5p_R+2	TGTAAACATCCCCGACTGGAAGCT	down	2,50E-03	high
8	hsa-miR-889-3p	TTAATATCGGACAACCATTGT	down	2,53E-03	middle
9	hsa-miR-654-3p_R-2	TATGTCTGCTGACCATCACC	down	2,63E-03	high
10	hsa-miR-193b-5p	CGGGGTTTTGAGGGCGAGATGA	up	2,67E-03	middle
11	hsa-miR-483-5p	AAGACGGGAGGAAAGAAGGGAG	up	3,17E-03	high
12	hsa-miR-99b-3p_R+2	CAAGCTCGTGTCTGTGGGTCCGTT	down	3,55E-03	middle
13	hsa-miR-329-3p	AACACACCTGGTTAACCTCTTT	down	3,56E-03	middle
14	hsa-miR-376c-3p	AACATAGAGGAAATTCCACGT	down	3,87E-03	middle
15	hsa-miR-338-5p_R-1	AACAATATCCTGGTGCTGAGT	down	4,34E-03	middle
16	hsa-miR-194-5p_R-1	TGTAACAGCAACTCCATGTGG	up	4,58E-03	middle
17	hsa-miR-151a-3p	CTAGACTGAAGCTCCTTGAGG	down	4,97E-03	high
18	hsa-miR-655-3p	ATAATACATGGTTAACCTCTTT	down	5,08E-03	middle
19	hsa-miR-323b-3p_R-1	CCCAATACACGGTCGACCTCT	down	5,24E-03	middle
20	hsa-miR-432-3p	CTGGATGGCTCCTCCATGTCT	down	5,72E-03	middle
21	hsa-miR-133a-3p_R+1	TTTGGTCCCCTTCAACCAGCTGT	down	6,44E-03	high
22	hsa-miR-1260a_R+3_1ss9TG	ATCCCACCGCTGCCACCAAAA	down	6,45E-03	middle
23	hsa-miR-369-3p	AATAATACATGGTTGATCTTT	down	6,48E-03	middle
24	hsa-miR-337-5p_R-1	GAACGGCTTCATACAGGAGT	down	6,69E-03	middle
25	hsa-miR-136-3p	CATCATCGTCTCAAATGAGTCT	down	6,75E-03	middle

26	hsa-miR-539-3p	ATCATACAAGGACAATTTCTTT	down	6,88E-03	middle
27	hsa-miR-126-5p	CATTATTACTTTTGGTACGCG	down	7,28E-03	high
28	hsa-miR-323a-3p_L+1	GCACATTACACGGTCGACCTCT	down	7,38E-03	middle
29	hsa-miR-11401_R+1	TCACGTCTGCGGCTGTCACGT	down	8,05E-03	middle
30	hsa-miR-7848-3p_L+2R-3	TGCTACCCTCGGTCTGCTTACC	down	8,83E-03	low
31	hsa-miR-361-5p	TTATCAGAATCTCCAGGGGTAC	down	9,50E-03	high
32	hsa-miR-411-3p_R-1	TATGTAACACGGTCCACTAAC	down	1,02E-02	middle
33	hsa-miR-1-3p	TGGAATGTAAAGAAGTATGTAT	down	1,07E-02	high
34	hsa-miR-382-3p_R+1	AATCATTCACGGACAACACTTT	down	1,14E-02	middle
35	hsa-miR-656-3p	AATATTATACAGTCAACCTCT	down	1,15E-02	middle
36	hsa-miR-410-3p	AATATAACACAGATGGCCTGT	down	1,18E-02	middle
37	hsa-miR-494-3p_R+1	TGAAACATACACGGGAAACCTCT	down	1,30E-02	high
38	hsa-miR-192-5p	CTGACCTATGAATTGACAGCC	up	1,36E-02	high
39	hsa-miR-381-3p	TATACAAGGGCAAGCTCTCTGT	down	1,45E-02	middle
40	hsa-miR-495-3p	AAACAAACATGGTGCACTTCTT	down	1,50E-02	middle
41	hsa-miR-376a-3p	ATCATAGAGGAAAATCCACGT	down	1,52E-02	middle
42	hsa-miR-4286_R+1	ACCCCACTCCTGGTACCA	down	1,52E-02	middle
43	hsa-miR-181c-3p_L-1R+1	ACCATCGACCGTTGAGTGGACC	down	1,65E-02	middle
44	hsa-miR-181d-3p_1ss21CT	CCACCGGGGGATGAATGTCAT	down	1,66E-02	low
45	hsa-miR-26b-3p_R+2	CCTGTTCTCCATTACTTGGCTCT	down	1,66E-02	middle
46	hsa-miR-340-5p	TTATAAAGCAATGAGACTGATT	down	1,69E-02	high
47	hsa-miR-409-3p	GAATGTTGCTCGGTGAACCCCT	down	1,76E-02	high
48	hsa-miR-382-5p	GAAGTTGTTCGTGGTGGATTCG	down	1,78E-02	middle
49	hsa-miR-484	TCAGGCTCAGTCCCCTCCCGAT	down	1,85E-02	high
50	hsa-miR-335-3p	TTTTTCATTATTGCTCCTGACC	down	2,01E-02	middle
51	hsa-miR-154-3p	AATCATACACGGTTGACCTATT	down	2,18E-02	middle
52	hsa-miR-335-5p	TCAAGAGCAATAACGAAAAATGT	down	2,20E-02	high
53	hsa-miR-4787-3p_R-1	GATGCGCCGCCCACTGCCCCGCG	down	2,27E-02	middle
54	hsa-miR-493-5p	TTGTACATGGTAGGCTTTCATT	down	2,32E-02	middle
55	hsa-miR-627-3p	TCTTTTCTTTGAGACTCACT	down	2,43E-02	middle
56	hsa-miR-299-3p	TATGTGGGATGGTAAACCGCTT	down	2,49E-02	middle
57	hsa-miR-6767-5p	TCGCAGACAGGGACACATGGAGA	down	2,51E-02	middle
58	hsa-miR-3065-3p_R-1	TCAGCACCAGGATATTGTTGGA	down	2,54E-02	low
59	hsa-miR-93-3p_R+1	ACTGCTGAGCTAGCACTTCCCGA	up	2,57E-02	middle
60	hsa-miR-204-5p	TTCCCTTTGTCATCCTATGCCT	down	2,60E-02	high
61	hsa-miR-3150a-5p	CAACCTCGACGATCTCCTCAGC	down	2,61E-02	middle
62	hsa-miR-625-3p	GACTATAGAACTTTCCCCCTCA	down	2,75E-02	middle
63	hsa-miR-219a-1-3p_R+1	AGAGTTGAGTCTGGACGTCCCGA	down	2,81E-02	middle
64	hsa-miR-193a-5p	TGGGTCTTTGCGGGCGAGATGA	up	2,84E-02	high
65	hsa-miR-211-5p_1ss18CT	TTCCCTTTGTCATCCTTTGCCT	down	2,86E-02	middle
66	hsa-miR-584-5p_R-1	TTATGGTTTGCCTGGGACTGA	down	2,88E-02	high
67	hsa-miR-409-5p	AGGTTACCCGAGCAACTTTGCAT	down	2,89E-02	middle
68	hsa-miR-133b_R-1	TTTGGTCCCCTTCAACCAGCT	down	3,03E-02	middle

69	hsa-miR-6876-5p	CAGGAAGGAGACAGGCAGTTCA	down	3,18E-02	middle
70	hsa-miR-485-3p	GTCATACACGGCTCTCCTCTCT	down	3,33E-02	middle
71	hsa-mir-4647-p5	ATGGTGCTGTGCTGAGGAAAGG	down	3,42E-02	low
72	hsa-miR-184	TGGACGGAGAACTGATAAGGGT	down	3,43E-02	high
73	hsa-miR-376c-5p_L-1R+1	GTGGATATTCCTTCTATGTTT	down	3,48E-02	low
74	hsa-miR-376b-5p_L-1	GTGGATATTCCTTCTATGTTT	down	3,48E-02	low
75	hsa-miR-151b_R+3	TCGAGGAGCTCACAGTCTAGT	down	3,54E-02	high
76	hsa-miR-1197	TAGGACACATGGTCTACTTCT	down	3,70E-02	middle
77	hsa-miR-3939_R-1	TACGCGCAGACCACAGGATGT	down	3,72E-02	low
78	hsa-miR-487b-3p	AATCGTACAGGGTCATCCACTT	down	3,76E-02	middle
79	hsa-miR-31-3p_R+1	TGCTATGCCAACATATTGCCATC	down	3,77E-02	middle
80	hsa-miR-486-3p	CGGGGCAGCTCAGTACAGGAT	up	3,78E-02	middle
81	hsa-miR-26a-1-3p	CCTATTCTTGGTTACTTGCACG	down	3,81E-02	middle
82	hsa-miR-377-5p	AGAGGTTGCCCTTGGTGAATTC	down	4,04E-02	middle
83	hsa-miR-135a-5p	TATGGCTTTTTATTCCTATGTGA	down	4,05E-02	middle
84	hsa-miR-6516-3p	ATCATGTATGATACTGCAAACA	down	4,20E-02	middle
85	hsa-miR-425-3p	ATCGGGAATGTCGTGTCCGCCC	down	4,20E-02	high
86	hsa-miR-30d-3p	CTTTCAGTCAGATGTTTGCTGC	down	4,29E-02	middle
87	hsa-miR-539-5p	GGAGAAATTATCCTTGGTGTGT	down	4,42E-02	middle
88	hsa-miR-3181_R+2	ATCGGGCCCTCGGCGCCGGCC	down	4,48E-02	low
89	hsa-miR-1185-1-3p	ATATACAGGGGGAGACTCTTAT	down	4,65E-02	middle
90	hsa-miR-380-3p_R-1	TATGTAATATGGTCCACATCT	down	4,68E-02	middle
91	hsa-miR-11400_1ss20TC	TCGGCTGTGTATCTCTGTGCC	down	4,71E-02	middle
92	hsa-miR-1185-2-3p_R-3	ATATACAGGGGGAGACTCT	down	4,75E-02	middle
93	hsa-miR-190b-5p	TGATATGTTTGATATTGGGTTG	down	4,76E-02	middle

Table S3: Different expressed microRNAs in late PDAC vs. healthy controls

1	hsa-miR-151a-3p	CTAGACTGAAGCTCCTTGAGG	down	4,78E-07	high
2	hsa-miR-493-3p	TGAAGGTCTACTGTGTGCCAGG	down	9,02E-06	middle
3	hsa-miR-11400_1ss20TC	TCGGCTGTGTATCTCTGTGCC	down	1,70E-05	middle
4	hsa-miR-584-5p_R-1	TTATGGTTTGCCTGGGACTGA	down	1,72E-05	high
5	hsa-miR-151b_R+3	TCGAGGAGCTCACAGTCTAGT	down	2,79E-05	high
6	hsa-miR-30d-3p	CTTTCAGTCAGATGTTTGCTGC	down	6,87E-05	middle
7	hsa-miR-3120-3p_R+1	CACAGCAAGTGTAGACAGGCAT	down	7,53E-05	middle
8	hsa-miR-421	ATCAACAGACATTAATTGGGCGC	down	7,91E-05	middle
9	hsa-miR-27b-3p	TTCACAGTGGCTAAGTTCTGC	down	8,00E-05	high
10	hsa-miR-199b-3p_R-1	ACAGTAGTCTGCACATTGGTT	down	1,08E-04	high
11	hsa-miR-1260b_R+1_1ss9AG	ATCCCACCGCTGCCACCATT	down	1,75E-04	middle
12	hsa-miR-4286_R+1	ACCCCACTCCTGGTACCA	down	2,09E-04	middle
13	hsa-miR-126-5p	CATTATTACTTTTGGTACGCG	down	2,19E-04	high
14	hsa-miR-30d-5p_R+2	TGTAAACATCCCCGACTGGAAGCT	down	2,20E-04	high
15	hsa-miR-548ag_R+1_1ss4GA	AAAAGTAATTGTGGTTTCTGCC	down	2,51E-04	middle
16	hsa-miR-4645-3p_L+2R-1	CGAGACAGTAGTTCTTGCCTGGT	down	2,61E-04	middle

17	hsa-miR-30e-3p	CTTTCAGTCGGATGTTTACAGC	down	3,04E-04	middle
18	hsa-miR-329-3p	AACACACCTGGTTAACCTCTTT	down	3,19E-04	middle
19	hsa-miR-409-3p	GAATGTTGCTCGGTGAACCCCT	down	3,31E-04	high
20	hsa-miR-628-3p_L+1	TTCTAGTAAGAGTGGCAGTCGA	down	4,59E-04	middle
21	hsa-miR-323b-3p_R-1	CCCAATACACGGTCGACCTCT	down	4,83E-04	middle
22	hsa-miR-323a-3p_L+1	GCACATTACACGGTCGACCTCT	down	4,99E-04	middle
23	hsa-miR-654-3p_R-2	TATGTCTGCTGACCATCACC	down	5,61E-04	high
24	hsa-miR-27a-3p	TTCACAGTGGCTAAGTTCCGC	down	5,70E-04	high
25	hsa-miR-487a-5p	GTGGTTATCCCTGCTGTGTTCG	down	6,18E-04	middle
26	hsa-miR-582-3p	TAACTGGTTGAACAACTGAACC	down	6,44E-04	middle
27	hsa-miR-432-5p	TCTTGGAGTAGGTCATTGGGTGG	down	6,50E-04	high
28	hsa-miR-337-5p_R-1	GAACGGCTTCATACAGGAGT	down	6,78E-04	middle
29	hsa-miR-6842-5p	TGGGGGTGGTCTCTAGCCAAGG	down	7,23E-04	middle
30	hsa-miR-1260a_R+3_1ss9TG	ATCCCACCGCTGCCACCAAAA	down	7,52E-04	middle
31	hsa-miR-556-5p_R-1	GATGAGCTCATTGTAATATGA	down	7,89E-04	middle
32	hsa-miR-494-3p_R+1	TGAAACATACACGGGAAACCTCT	down	8,73E-04	high
33	hsa-miR-15b-5p	TAGCAGCACATCATGGTTTACA	down	8,81E-04	high
34	hsa-miR-1271-5p	CTTGGCACCTAGCAAGCACTCA	down	9,30E-04	middle
35	hsa-miR-3912-3p	TAACGCATAATATGGACATGT	down	9,39E-04	middle
36	hsa-miR-484	TCAGGCTCAGTCCCCTCCCGAT	down	1,01E-03	high
37	hsa-miR-6837-3p	CCTTCACTGTGACTCTGCTGCAG	down	1,03E-03	middle
38	hsa-miR-4677-3p	TCTGTGAGACCAAAGAACTACT	down	1,03E-03	middle
39	hsa-miR-376c-3p	AACATAGAGGAAATTCCACGT	down	1,22E-03	middle
40	hsa-miR-6852-5p	CCCTGGGGTTCTGAGGACATG	down	1,23E-03	middle
41	hsa-miR-889-3p	TTAATATCGGACAACCATTGT	down	1,35E-03	middle
42	hsa-miR-665_R+2	ACCAGGAGGCTGAGGCCCCTCT	down	1,39E-03	middle
43	hsa-miR-130a-3p	CAGTGCAATGTTAAAAGGGCAT	down	1,62E-03	middle
44	hsa-miR-130b-5p_R+1	ACTCTTTCCCTGTTGCACTACT	down	1,65E-03	middle
45	hsa-miR-191-3p_L-1	CTGCGCTTGGATTTCGTCCCC	down	1,67E-03	middle
46	hsa-miR-493-5p	TTGTACATGGTAGGCTTTCATT	down	1,79E-03	middle
47	hsa-miR-381-3p	TATACAAGGGCAAGCTCTCTGT	down	1,88E-03	middle
48	hsa-miR-181c-3p_L-1R+1	ACCATCGACCGTTGAGTGGACC	down	1,92E-03	middle
49	hsa-miR-26a-1-3p	CCTATTCTTGGTTACTTGCACG	down	1,94E-03	middle
50	hsa-miR-625-3p	GACTATAGAACTTTCCCCCTCA	down	1,95E-03	middle
51	hsa-miR-10399-5p	AATTACAGATTGTCTCAGAGA	down	2,01E-03	middle
52	hsa-miR-382-5p	GAAGTTGTTCGTGGTGGATTCG	down	2,11E-03	middle
53	hsa-miR-4662a-5p	TTAGCCAATTGTCCATCTTTAG	down	2,20E-03	middle
54	hsa-let-7e-3p	CTATACGGCCTCCTAGCTTTCC	down	2,20E-03	middle
55	hsa-miR-616-5p_R-1	ACTCAAAACCCTTCAGTGACT	down	2,31E-03	middle
56	hsa-miR-3120-5p_R-1	CCTGTCTGTGCCTGCTGTAC	down	2,32E-03	middle
57	hsa-miR-3065-5p_R+2	TCAACAAAATCACTGATGCTGGAGT	down	2,34E-03	middle
58	hsa-miR-361-5p	TTATCAGAATCTCCAGGGGTAC	down	2,45E-03	high
59	hsa-miR-425-3p	ATCGGGAATGTCGTGTCCGCCC	down	2,52E-03	high

60	hsa-let-7d-3p	CTATACGACCTGCTGCCTTTCT	down	2,67E-03	high
61	hsa-miR-539-3p	ATCATACAAGGACAATTTCTTT	down	2,69E-03	middle
62	hsa-mir-641-p3	TGACTGTCCTATGTCTTTCCT	down	2,76E-03	middle
63	hsa-miR-758-5p_L-1R+1	ATGGTTGACCAGAGAGCACACG	down	2,79E-03	middle
64	hsa-miR-543	AAACATTCGCGGTGCACTTCTT	down	2,88E-03	middle
65	hsa-miR-758-3p_R-1	TTTGTGACCTGGTCCACTAAC	down	2,97E-03	middle
66	hsa-miR-142-5p_L+2R-3	CCCATAAAGTAGAAAGCACT	down	3,36E-03	high
67	hsa-miR-26b-3p_R+2	CCTGTTCTCCATTACTTGGCTCT	down	3,38E-03	middle
68	hsa-miR-4701-5p	TTGGCCACCACACCTACCCCTT	down	3,48E-03	middle
69	hsa-miR-4676-3p_L-1	ACTGTTTCACCACTGGCTCTT	down	3,57E-03	middle
70	hsa-miR-3942-5p_L-1	AGCAATACTGTTACCTGAAAT	down	3,77E-03	middle
71	hsa-miR-3157-3p	CTGCCCTAGTCTAGCTGAAGCT	down	4,02E-03	middle
72	hsa-miR-16-1-3p	CCAGTATTAACTGTGCTGCTGA	down	4,26E-03	middle
73	hsa-miR-199a-5p	CCCAGTGTTCAGACTACCTGTTC	down	4,52E-03	high
74	hsa-miR-136-3p	CATCATCGTCTCAAATGAGTCT	down	4,73E-03	middle
75	hsa-miR-28-3p	CACTAGATTGTGAGCTCCTGGA	down	4,75E-03	high
76	hsa-miR-4448_1ss6CG	GGCTCGTTGGTCTAGGGGTA	down	4,86E-03	middle
77	hsa-miR-7-1-3p	CAACAAATCACAGTCTGCCATA	down	4,97E-03	middle
78	hsa-miR-654-5p	TGGTGGGCCGCAGAACATGTGC	down	4,97E-03	middle
79	hsa-miR-148b-5p_L+1	GAAGTTCTGTTATACACTCAGGC	down	4,97E-03	middle
80	hsa-miR-338-5p_R-1	AACAATATCCTGGTGCTGAGT	down	4,99E-03	middle
81	hsa-miR-7977_1ss6AG	TTCCCGGCCAACGCACCA	down	5,05E-03	middle
82	hsa-miR-377-5p	AGAGGTTGCCCTTGGTGAATTC	down	5,32E-03	middle
83	hsa-miR-3135a_R+1	TGCCTAGGCTGAGACTGCAGTGT	down	5,77E-03	middle
84	hsa-miR-128-1-5p_R-2	CGGGGCCGTAGCACTGTCTGA	down	5,81E-03	middle
85	hsa-miR-4433b-5p_R+1	ATGTCCCACCCCCACTCCTGTT	down	5,84E-03	high
86	hsa-miR-1185-2-3p_R-3	ATATACAGGGGGAGACTCT	down	5,88E-03	middle
87	hsa-mir-548v-p3_1ss6GA	CAAAAACTACAGTTACTTTTGC	down	5,98E-03	low
88	hsa-miR-548av-5p_R+4	AAAAGTACTTGCGGATTTTGCT	down	6,20E-03	middle
89	hsa-miR-7-5p_R-1	TGGAAGACTAGTGATTTTGTTGT	up	6,25E-03	middle
90	hsa-miR-7151-3p_L+1	CCTACAGGCTGGAATGGGCTCA	down	6,34E-03	low
91	hsa-miR-885-5p	TCCATTACACTACCCTGCCTCT	up	6,35E-03	middle
92	hsa-miR-376a-3p	ATCATAGAGGAAAATCCACGT	down	6,42E-03	middle
93	hsa-miR-6513-3p_R+1	TCAAGTGTCATCTGTCCCTAGA	down	6,47E-03	middle
94	hsa-miR-627-3p	TCTTTTCTTTGAGACTCACT	down	6,68E-03	middle
95	hsa-miR-335-5p	TCAAGAGCAATAACGAAAAATGT	down	6,70E-03	high
96	hsa-miR-6741-3p	TCGGCTCTCTCCCTCACCCTAG	down	6,92E-03	middle
97	hsa-miR-495-3p	AAACAAACATGGTGCACTTCTT	down	7,03E-03	middle
98	hsa-miR-152-3p	TCAGTGCATGACAGAACTTGG	down	7,11E-03	high
99	hsa-miR-616-3p_L+1	AAGTCATTGGAGGGTTTGAGCAG	down	7,13E-03	middle
100	hsa-miR-655-3p	ATAATACATGGTTAACCTCTTT	down	7,15E-03	middle
101	hsa-miR-22-3p	AAGCTGCCAGTTGAAGAACTGT	up	7,19E-03	high
102	hsa-miR-4688	TAGGGGCAGCAGAGGACCTGGG	down	7,19E-03	middle

103	hsa-miR-221-5p	ACCTGGCATACAATGTAGATTT	down	7,30E-03	middle
104	hsa-miR-382-3p_R+1	AATCATTCACGGACAACACTTT	down	7,40E-03	middle
105	hsa-miR-744-3p_R-1	CTGTTGCCACTAACCTCAACC	down	7,48E-03	middle
106	hsa-mir-3648-2-p3_1ss23CT	CCCCGCGCGTGTGTCCCGGCTGT	down	7,58E-03	low
107	hsa-miR-301a-5p	GCTCTGACTTTATTGCACTACT	down	7,81E-03	middle
108	hsa-miR-548e-5p	CAAAAGCAATCGCGGTTTTTGC	down	8,13E-03	middle
109	hsa-miR-1304-3p_1ss13CA	TCTCACTGTAGCATCGAACCCC	down	8,36E-03	middle
110	hsa-miR-656-3p	AATATTATACAGTCAACCTCT	down	8,61E-03	middle
111	hsa-miR-411-3p_R-1	TATGTAACACGGTCCACTAAC	down	8,77E-03	middle
112	hsa-miR-7848-3p_L+2R-3	TGCTACCCTCGGTCTGCTTACC	down	8,83E-03	low
113	hsa-miR-4775	TTAATTTTTTGTTTCGGTCACT	down	8,92E-03	low
114	hsa-miR-337-3p_L-1	TCCTATATGATGCCTTTCTTC	down	9,01E-03	middle
115	hsa-miR-6876-5p	CAGGAAGGAGACAGGCAGTTCA	down	9,06E-03	middle
116	hsa-miR-4659a-3p	TTTCTTCTTAGACATGGCAACG	down	9,13E-03	middle
117	hsa-miR-664b-5p_R-1	TGGGCTAAGGGAGATGATTGGGT	down	9,14E-03	middle
118	hsa-mir-937-p3	CCCATCCGCGCTCTGACTCT	down	9,56E-03	middle
119	hsa-miR-548au-5p_L-1R+1	AAAGTAATTGCGGTTTTTGCC	down	9,79E-03	middle
120	hsa-miR-98-3p_1ss22CT	CTATACAACTTACTACTTTCCT	down	9,87E-03	middle
121	hsa-miR-340-5p	TTATAAAGCAATGAGACTGATT	down	9,91E-03	high
122	hsa-miR-1301-3p	TTGCAGCTGCCTGGGAGTGACTTC	down	1,00E-02	middle
123	hsa-miR-134-3p_L-1R-1	CTGTGGGCCACCTAGTCACCA	down	1,04E-02	low
124	hsa-miR-485-5p_R+1	AGAGGCTGGCCGTGATGAATTCG	down	1,04E-02	middle
125	hsa-miR-6772-3p_1ss23AT	TTGCTCCTGACTCTGTGCCCACT	down	1,05E-02	middle
126	hsa-mir-4454-p5_1ss19AT	TCCGAGTCACGGCACCAAT	down	1,07E-02	middle
127	hsa-miR-6516-3p	ATCATGTATGATACTGCAAACA	down	1,07E-02	middle
128	hsa-miR-1468-5p_R+1	CTCCGTTTGCCTGTTTCGCTGA	down	1,07E-02	middle
129	hsa-miR-219b-5p	AGATGTCCAGCCACAATTCTCG	down	1,09E-02	middle
130	hsa-miR-4687-3p	TGGCTGTTGGAGGGGGCAGGC	down	1,11E-02	middle
131	hsa-miR-5010-3p_R+1	TTTTGTGTCTCCCATTCCCCAGT	down	1,15E-02	middle
132	hsa-miR-550b-3p_L+1R+1	CTCTTACTCCCTCAGGCACTGC	down	1,17E-02	low
133	hsa-miR-556-3p_R-1	ATATTACCATTAGCTCATCTT	down	1,18E-02	middle
134	hsa-miR-3065-3p_R-1	TCAGCACCAGGATATTGTTGGA	down	1,25E-02	low
135	hsa-miR-380-5p_L+1R-1	ATGGTTGACCATAGAACATGCG	down	1,27E-02	middle
136	hsa-miR-496_L-2R+1	AGTATTACATGGCCAATCTCT	down	1,28E-02	middle
137	hsa-miR-6818-5p	TTGTGTGAGTACAGAGAGCATC	down	1,29E-02	middle
138	hsa-miR-485-3p	GTCATACACGGCTCTCCTCTCT	down	1,30E-02	middle
139	hsa-miR-548l_R-1	AAAAGTATTTGCGGGTTTTGT	down	1,35E-02	middle
140	hsa-miR-576-5p	ATTCTAATTTCTCCACGTCTTT	down	1,35E-02	middle
141	hsa-miR-1908-3p_R+1	CCGGCCGCCGGCTCCGCCCCGT	down	1,36E-02	middle
142	hsa-miR-409-5p	AGGTTACCCGAGCAACTTTGCAT	down	1,36E-02	middle
143	hsa-miR-32-3p_R-1	CAATTTAGTGTGTGTGATATT	down	1,45E-02	middle
144	hsa-miR-605-3p	AGAAGGCACTATGAGATTTAGA	down	1,46E-02	middle
145	hsa-mir-548ag-2-p3	CAAGAACCTCAATTACCTTTGC	down	1,50E-02	middle

146	hsa-miR-598-3p	TACGTCATCGTTGTCATCGTCA	down	1,50E-02	middle
147	hsa-miR-628-5p	ATGCTGACATATTTACTAGAGG	down	1,50E-02	middle
148	hsa-mir-1256-p3	CTAAAGAGAAGTCAATGCATGA	down	1,53E-02	middle
149	hsa-miR-192-5p	CTGACCTATGAATTGACAGCC	up	1,55E-02	high
150	hsa-miR-18a-5p_R-3	TAAGGTGCATCTAGTGCAGA	up	1,56E-02	middle
151	hsa-miR-548o-3p	CCAAAACTGCAGTTACTTTTGC	down	1,58E-02	middle
152	hsa-miR-3934-5p	TCAGGTGTGGAAACTGAGGCAG	down	1,60E-02	middle
153	hsa-miR-182-3p_R-1	TGGTTCTAGACTTGCCAACT	down	1,70E-02	middle
154	hsa-miR-1226-3p	TCACCAGCCCTGTGTTCCCTAG	down	1,71E-02	middle
155	hsa-miR-369-3p	AATAATACATGGTTGATCTTT	down	1,72E-02	middle
156	hsa-miR-362-5p_R-2	AATCCTTGGAACCTAGGTGTGA	up	1,72E-02	middle
157	hsa-mir-1976-p5_1ss22CT	GCAGCAAGGAAGGCAGGGGTCT	down	1,74E-02	middle
158	hsa-miR-4654	TGTGGGATCTGGAGGCATCTGG	down	1,74E-02	middle
159	hsa-miR-134-5p	TGTGACTGGTTGACCAGAGGGG	down	1,74E-02	high
160	hsa-miR-18a-3p_R-2	ACTGCCCTAAGTGCTCCTTCT	up	1,78E-02	middle
161	hsa-miR-106b-3p_R-2	CCGCACTGTGGGTACTTGCT	up	1,80E-02	high
162	hsa-miR-24-3p_R-2	TGGCTCAGTTCAGCAGGAAC	down	1,80E-02	high
163	hsa-miR-487a-3p	AATCATACAGGGACATCCAGTT	down	1,83E-02	middle
164	hsa-miR-548h-3p_R-1_1ss4AG	CAAGAACCGCAATTACTTTTGC	down	1,87E-02	middle
165	hsa-miR-410-3p	AATATAACACAGATGGCCTGT	down	1,87E-02	middle
166	hsa-miR-181b-3p_R-1	CTCACTGAACAATGAATGCA	down	1,92E-02	middle
167	hsa-miR-548am-3p_R-2	CAAAAACTGCAGTTACTTTT	down	1,99E-02	low
168	hsa-miR-577_L+1	GTAGATAAAATATTGGTACCTG	down	2,00E-02	middle
169	hsa-miR-497-5p	CAGCAGCACACTGTGGTTTGT	up	2,07E-02	middle
170	hsa-mir-548h-2-p3	ACCACAATTACTTTTGCACCA	down	2,09E-02	low
171	hsa-let-7a-3p_R+1	CTATACAATCTACTGTCTTTCT	down	2,09E-02	middle
172	hsa-miR-3617-5p	AAAGACATAGTTGCAAGATGGG	down	2,14E-02	middle
173	hsa-miR-3143_R-4	ATAACATTGTAAAGCGCTTCT	down	2,14E-02	middle
174	hsa-miR-374b-5p_R-1	ATATAATACAACCTGCTAAGT	down	2,22E-02	middle
175	hsa-miR-590-3p	TAATTTTATGTATAAGCTAGT	down	2,24E-02	middle
176	hsa-miR-5006-3p_R+1	TTTCCCTTTCCATCCTGGCAGA	down	2,25E-02	middle
177	hsa-miR-99b-3p_R+2	CAAGCTCGTGTCTGTGGGTCCGTT	down	2,25E-02	middle
178	hsa-miR-335-3p	TTTTTCATTATTGCTCCTGACC	down	2,29E-02	middle
179	hsa-miR-5001-3p_R-1	TTCTGCCTCTGTCCAGGTCCT	down	2,30E-02	middle
180	hsa-miR-11401_R+1	TCACGTCTGCGGCTGTCACGT	down	2,30E-02	middle
181	hsa-miR-433-3p	ATCATGATGGGCTCCTCGGTGT	down	2,31E-02	middle
182	hsa-miR-652-3p_R+1	AATGGCGCCACTAGGGTTGTGT	down	2,32E-02	high
183	hsa-miR-21-3p	CAACACCAGTCGATGGGCTGT	down	2,33E-02	high
184	hsa-miR-452-5p_L-3R+2	TGTTTGCAGAGGAAACTGAGA	up	2,34E-02	middle
185	hsa-miR-197-3p	TTCACCACCTTCTCCACCCAGC	down	2,35E-02	middle
186	hsa-miR-450a-2-3p_L-1	TTGGGGACATTTTGCATTCAT	down	2,36E-02	middle
187	hsa-miR-374b-3p	CTTAGCAGGTTGTATTATCATT	down	2,39E-02	middle
188	hsa-miR-4672	TTACACAGCTGGACAGAGGCA	down	2,43E-02	middle

189	hsa-miR-20b-5p	CAAAGTGCTCATAGTGCAGGTAG	up	2,44E-02	middle
190	hsa-miR-5584-5p	CAGGGAAATGGGAAGAACTAGA	down	2,44E-02	low
191	hsa-miR-4498_R-1	TGGGCTGGCAGGGCAAGTGCT	down	2,46E-02	low
192	hsa-miR-4743-5p_R-2	TGGCCGGATGGGACAGGAGGC	down	2,54E-02	middle
193	hsa-miR-454-5p	ACCCTATCAATATTGTCTCTGC	down	2,55E-02	middle
194	hsa-miR-561-5p	ATCAAGGATCTTAAACTTTGCC	down	2,56E-02	middle
195	hsa-miR-136-5p_R-1	ACTCCATTTGTTTTGATGATGG	down	2,62E-02	middle
196	hsa-miR-433-5p_R-1	TACGGTGAGCCTGTCATTATT	down	2,63E-02	middle
197	hsa-miR-6736-5p	CTGGGTGAGGGCATCTGTGGT	down	2,67E-02	middle
198	hsa-miR-1914-5p_R+1	CCCTGTGCCCGGCCCACTTCTGT	down	2,69E-02	middle
199	hsa-miR-26a-5p	TTCAAGTAATCCAGGATAGGCT	down	2,70E-02	high
200	hsa-miR-548u_R-1	CAAAGACTGCAATTACTTTTGC	down	2,72E-02	middle
201	hsa-miR-1197	TAGGACACATGGTCTACTTCT	down	2,72E-02	middle
202	hsa-miR-6813-5p_R-1	CAGGGGCTGGGGTTTCAGGTTC	down	2,75E-02	middle
203	hsa-miR-6882-5p_L-1R+1	ACAAGTCAGGAGCTGAAGCAGC	down	2,75E-02	middle
204	hsa-miR-4732-3p_R-1	GCCCTGACCTGTCCTGTTCT	up	2,76E-02	middle
205	hsa-miR-151b_R+2	TCGAGGAGCTCACAGTCTAG	down	2,77E-02	middle
206	hsa-miR-3140-3p_R-1	AGCTTTTGGGAATTCAGGTAG	down	2,77E-02	middle
207	hsa-miR-377-3p	ATCACACAAAGGCAACTTTTGT	down	2,79E-02	middle
208	hsa-miR-320a-3p	AAAAGCTGGGTTGAGAGGGCGA	up	2,82E-02	high
209	hsa-miR-3177-3p	TGCACGGCACTGGGGACACGT	down	2,85E-02	middle
210	hsa-miR-5189-3p	TGCCAACCGTCAGAGCCCAGA	down	2,85E-02	middle
211	hsa-miR-5683	TACAGATGCAGATTCTCTGACTTC	down	2,94E-02	middle
212	hsa-miR-3136-5p_R-1	CTGACTGAATAGGTAGGGTCAT	down	2,97E-02	low
213	hsa-miR-4767_L+1R-5	CCGCGGGCGCTCCTGGCCG	down	2,99E-02	middle
214	hsa-miR-331-3p_L-1R+1	CCCCTGGGCCTATCCTAGAAT	down	2,99E-02	middle
215	hsa-miR-766-3p	ACTCCAGCCCCACAGCCTCAGC	down	3,02E-02	middle
216	hsa-miR-2355-5p_R+1	ATCCCCAGATACAATGGACAAT	down	3,05E-02	middle
217	hsa-mir-3138-p5	ACTTCCCCCACCTCACTGCCC	down	3,05E-02	middle
218	hsa-miR-539-5p	GGAGAAATTATCCTTGGTGTGT	down	3,06E-02	middle
219	hsa-miR-324-3p_L-3R+1	ACTGCCCCAGGTGCTGCTGGA	up	3,06E-02	middle
220	hsa-miR-487b-3p	AATCGTACAGGGTCATCCACTT	down	3,12E-02	middle
221	hsa-miR-548ac_R-1	CAAAAACCGGCAATTACTTTT	down	3,15E-02	low
222	hsa-miR-664a-3p	TATTCATTTATCCCCAGCCTACA	down	3,18E-02	middle
223	hsa-miR-659-5p	AGGACCTTCCCTGAACCAAGGA	down	3,23E-02	middle
224	hsa-miR-122-5p_R-1	TGGAGTGTGACAATGGTGTTT	up	3,31E-02	high
225	hsa-miR-194-5p_R-1	TGTAACAGCAACTCCATGTGG	up	3,36E-02	middle
226	hsa-miR-93-3p_R+1	ACTGCTGAGCTAGCACTTCCCGA	up	3,41E-02	middle
227	hsa-miR-592	TTGTGTCAATATGCGATGATGT	down	3,43E-02	middle
228	hsa-miR-210-3p	CTGTGCGTGTGACAGCGGCTGA	up	3,45E-02	middle
229	hsa-miR-1275_R+1	GTGGGGGAGAGGCTGTCG	down	3,58E-02	middle
230	hsa-miR-6859-3p_L+2R-3	CCTGACCCCCATGTCGCCTCTG	down	3,59E-02	low
231	hsa-miR-379-5p	TGGTAGACTATGGAACGTAGG	down	3,59E-02	middle

232	hsa-miR-1185-1-3p	ATATACAGGGGGAGACTCTTAT	down	3,62E-02	middle
233	hsa-mir-1302-1-p5_1ss9AG	GAATTTCAGGTAAACAATGAAT	down	3,63E-02	low
234	hsa-miR-1296-5p	TTAGGGCCCTGGCTCCATCTCC	down	3,64E-02	middle
235	hsa-miR-3939_R-1	TACGCGCAGACCACAGGATGT	down	3,72E-02	low
236	hsa-miR-329-5p	GAGGTTTTCTGGGTTTCTGTTTC	down	3,76E-02	low
237	hsa-miR-222-5p_R-1	CTCAGTAGCCAGTGTAGATCC	down	3,89E-02	middle
238	hsa-miR-4687-5p_R-1	CAGCCCTCCTCCCGCACCCAA	down	3,91E-02	middle
239	hsa-miR-193a-3p_R-2	AACTGGCCTACAAAGTCCCA	down	3,93E-02	middle
240	hsa-miR-1228-5p	GTGGGCGGGGGCAGGTGTGTG	up	3,95E-02	middle
241	hsa-miR-16-5p	TAGCAGCACGTAAATATTGGCG	down	3,97E-02	high
242	hsa-miR-494-5p_R-1	AGGTTGTCCGTGTTGTCTTCTC	down	4,03E-02	low
243	hsa-mir-548ax-p3	CAAAAACCGTAATTACTTTTGT	down	4,05E-02	middle
244	hsa-miR-188-3p	CTCCCACATGCAGGGTTTGCA	down	4,06E-02	middle
245	hsa-miR-320e_L+1R+3_1ss17AG	AAAAGCTGGGTTGAGAGGGTGA	up	4,12E-02	middle
246	hsa-mir-548ap-p5_1ss2AG	TGGGTTGGTGCAAAAGTAATTG	down	4,13E-02	low
247	hsa-mir-603-p5_1ss4TG	TGGGTTGGTGCAAAAGTAATTG	down	4,13E-02	low
248	hsa-miR-185-5p	TGGAGAGAAAGGCAGTTCCTGA	up	4,14E-02	high
249	hsa-miR-125b-5p	TCCCTGAGACCCTAACTTGTGA	up	4,14E-02	high
250	hsa-miR-541-3p	TGGTGGGCACAGAATCTGGACT	down	4,24E-02	middle
251	hsa-miR-92a-3p	TATTGCACTTGTCCCGGCCTGT	down	4,32E-02	high
252	hsa-miR-7849-3p	GACAATTGTTGATCTTGGGCCT	down	4,32E-02	middle
253	hsa-miR-6779-5p_L-1	TGGGAGGGGCTGGGTTTGGC	down	4,32E-02	middle
254	hsa-miR-4422_R+1	AAAAGCATCAGGAAGTACCCAC	down	4,35E-02	middle
255	hsa-miR-215-5p_R+1	ATGACCTATGAATTGACAGACA	up	4,39E-02	middle
256	hsa-miR-3181_R+2	ATCGGGCCCTCGGCGCCGGCC	down	4,48E-02	low
257	hsa-miR-3920	ACTGATTATCTTAACTCTCTGA	down	4,49E-02	low
258	hsa-miR-1228-3p_R+1	TCACACCTGCCTCGCCCCCCA	up	4,49E-02	middle
259	hsa-miR-154-3p	AATCATACACGGTTGACCTATT	down	4,50E-02	middle
260	hsa-miR-19b-3p_R-2	TGTGCAAATCCATGCAAAACT	up	4,53E-02	high
261	hsa-miR-3127-3p_R+1	TCCCCTTCTGCAGGCCTGCTGGA	down	4,56E-02	middle
262	hsa-miR-26b-5p_R+1	TTCAAGTAATTCAGGATAGGTT	down	4,68E-02	high
263	hsa-miR-3074-3p_L+1R+1	TGATATCAGCTCAGTAGGCACCGT	down	4,69E-02	middle
264	hsa-miR-3944-5p	TGTGCAGCAGGCCAACCGAGA	down	4,70E-02	middle
265	hsa-miR-376b-3p	ATCATAGAGGAAAATCCATGTT	down	4,71E-02	middle
266	hsa-miR-3677-3p	CTCGTGGGCTCTGGCCACGGCC	down	4,84E-02	middle
267	hsa-miR-5188_L-1R+1	ATCGGACCCATTTAAACCGGAGA	down	4,85E-02	low
268	hsa-miR-371b-5p	ACTCAAAAGATGGCGGCACTTT	down	4,87E-02	middle
269	hsa-miR-6799-5p_L+1R+1	AGGGGAGGTGTGCAGGGCTGGT	down	4,89E-02	middle
270	hsa-miR-744-5p_R-1	TGCGGGGCTAGGGCTAACAGC	down	4,95E-02	high
271	hsa-miR-299-3p	TATGTGGGATGGTAAACCGCTT	down	4,99E-02	middle
